# Aggressive variant of splenic marginal zone lymphoma characterized using a cancer panel test and treated with rituximab-containing chemotherapy: A case report

**DOI:** 10.1097/MD.0000000000021938

**Published:** 2020-08-28

**Authors:** Kazuya Ishiguro, Yasushi Sasaki, Yoshitake Takagi, Takeshi Niinuma, Hiromu Suzuki, Takashi Tokino, Toshiaki Hayashi, Tohru Takahashi, Tetsuyuki Igarashi, Yoshihiro Matsuno

**Affiliations:** a Department of Hematology, Tenshi Hospital; b Department of Molecular Biology, Sapporo Medical University School of Medicine; c Center for Medical Education, Sapporo Medical University; d Pathology Center, Genetic Lab Co., Ltd; e Department of Medical Genome Sciences, Research Institute for Frontier Medicine, Sapporo Medical University School of Medicine; f Department of Hematology, Teine Keijinkai Hospital, Sapporo, Japan.; g Department of Surgical Pathology, Hokkaido University Hospital.

**Keywords:** aggressive variant of splenic marginal zone lymphoma, cancer panel test, next generation sequencing, rituximab

## Abstract

**Rationale:**

Aggressive variant of splenic marginal zone lymphoma (AV-SMZL) is a very rare disease that is often associated with *TP53* mutations and has a poor prognosis. On the other hand, recent advances in genome sequencing techniques enable us to understand the molecular characteristics of rare cancers such as AV-SMZL. Here we present a case of AV-SMZL analyzed using a genetic test.

**Patient Concerns:**

A 66-year-old woman was admitted with splenomegaly and lymphocytosis. Computed tomography revealed marked splenomegaly without lymphadenopathy in any other areas. The serum soluble interleukin-2 receptor (sIL-2R) level was significantly elevated. Peripheral and bone marrow blood tests showed an increase in abnormal lymphocytes.

**Diagnosis:**

A splenectomy revealed an SMZL pattern with increased numbers of large cells and mitotic cells and a high Ki-67 positivity rate, which led to a diagnosis of AV-SMZL. Although *TP53* mutation was not detected, mutations in *NOTCH2*, *NCOA4*, *PTEN*, *EPHA3,* and *KMT2D* were identified. Among these, the mutations in *NCOA4*, *PTEN,* and *EPHA3* were novel pathogenic mutations in SMZL, which suggests they may be related to the aggressiveness and persistence of the disease.

**Interventions:**

The patient was administered a rituximab-containing regimen and rituximab-maintenance therapy.

**Outcomes:**

The patient continues to exhibit a complete response.

**Lessons:**

This is a case of AV-SMZL in which a cancer panel test successfully detected genetic alterations that are potentially associated with its pathogenesis. These findings suggest that genetic analysis is useful for making diagnoses as well as for determining treatment strategies in AV-SMZL.

## 1 Introduction

Aggressive variant of splenic marginal zone lymphoma (AV-SMZL) is a rare disorder. Although little is known about its pathogenesis or clinical characteristics, earlier studies reported that AV-SMZL is associated with an increase in large abnormal lymphocytes, with*TP53* mutations, and with an aggressive disease course and poor prognosis.^[1–5]^ In recent years, cancer panel tests using next-generation sequencing technologies have emerged as powerful tools for diagnosing rare or complex neoplasms.^[6]^ Here we present a case of AV-SMZL that exhibited mutations in multiple cancer-related genes, including *NOTCH2*, *NCOA4*, *PTEN*, *EPHA3,* and *KMT2D*. The information we obtained from this case may be helpful for establishing accurate diagnosis and better management of AV-SMZL.

## 2 Methods

### 2.1 Clinical specimens

Specimens of spleen and peripheral blood were collected from the patient. Genomic DNA was extracted using QIAamp DNA Mini Kits (Qiagen, Hilden, Germany) according to the manufacturer's instructions. This study was performed in accordance with the Declaration of Helsinki and was approved by the Institutional Review Boards of Sapporo Medical University and Tenshi Hospital. Written informed consent was obtained from the patient for publication of this case report and accompanying images.

### 2.2 Mutation analysis

For mutation analysis of the specimens of spleen, targeted sequencing of 409 cancer-related genes was performed using an Ion Ampliseq Comprehensive Cancer Panel (Thermo Fisher Scientific, Waltham, MA) and an Ion Proton System (Thermo Fisher Scientific) as previously described.^[7]^ Nucleotide variants were detected using Ion Reporter (Thermo Fisher Scientific). Leukocytes in peripheral blood after a complete response (CR) served as a normal control. FATHMM-XF was used for predicting the functions of the mutations.^[8]^

## 3 Case report

**Figure 1:**
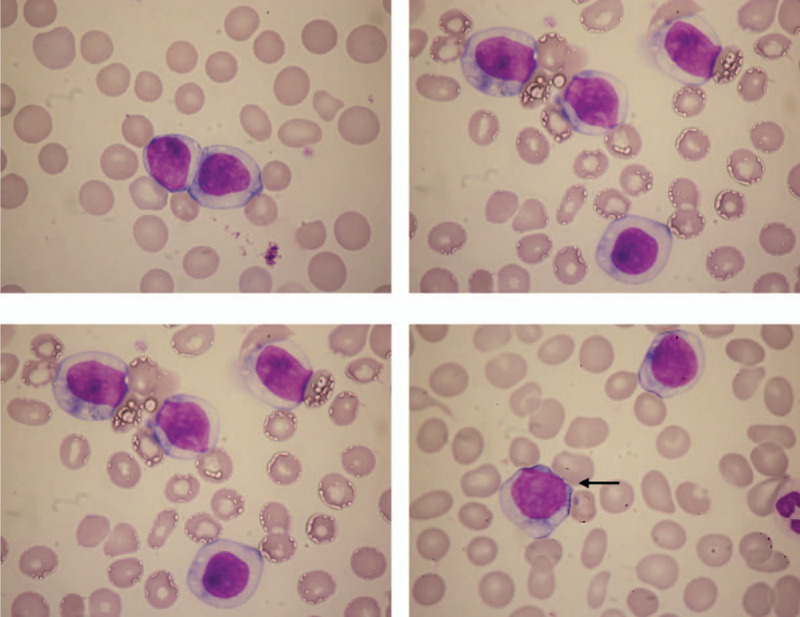
Increased numbers of lymphoma cells in the peripheral blood. The cell indicated by the arrow had a moderately visible nucleolus. [May–Giemsa staining (1000×)].

**Figure 2:**
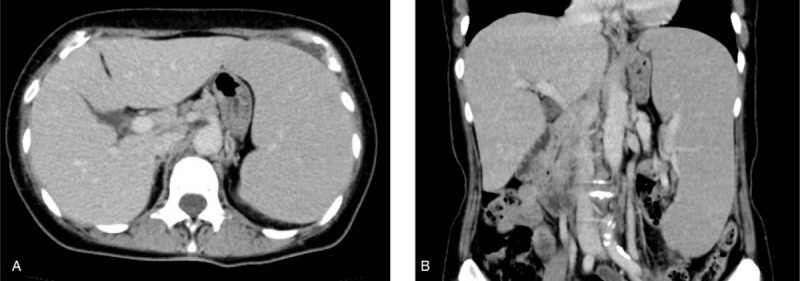
Computed tomography of the abdomen showing splenomegaly. (A) Horizontal axis. (B) Coronal axis.

**Figure 3:**
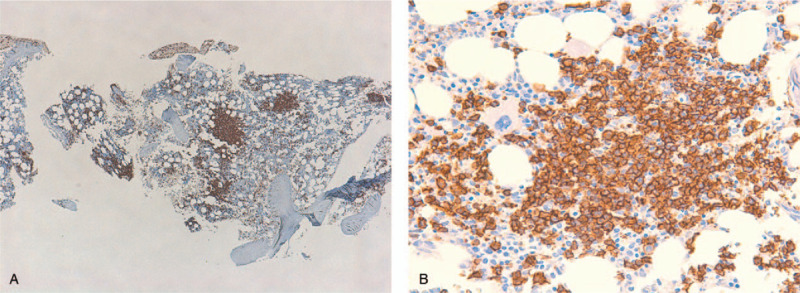
Partial aggregation of B-lymphocytes in bone marrow. (A) CD20 staining (40×). (B) CD20 staining (400×).

A 66-year-old woman was admitted to our hospital for treatment of lymphoma. She had been healthy until 1 month before this admission, when she developed pain in her left leg. Although the precise reason of the pain was unknown, splenomegaly and lymphocytosis were found. A physical examination showed splenomegaly without superficial lymphadenopathy or hepatomegaly. Peripheral blood showed an increase in medium-sized mature lymphocyte-like cells. They had abundant, moderately basophilic cytoplasm and moderately condensed chromatin without villous projections. Approximately 22% of the lymphocytes in her peripheral blood had slightly visible nucleoli (Fig. 1). Anemia and thrombocytopenia were present (Table 1). Laboratory data showed significant elevation of serum soluble interleukin-2 receptor (sIL-2R), while lactic acid dehydrogenase (LDH) was within the normal range (Table 1). Computed tomography revealed marked splenomegaly without lymphadenopathy in any other areas (Fig. 2A and B). Bone marrow aspirate also showed an increase in similar lymphocyte-like cells. However, hematopoiesis of 3 lineages remained normal in the bone marrow. This made it difficult to confirm a diagnosis based on peripheral and bone marrow blood findings, as the cell surface markers indicated a confusing mixture of B cell and T cell lineages, and the bone marrow biopsy findings showed only partial aggregation of B-lymphocytes and were not definitive (Fig. 3A and B).

**Table 1:**
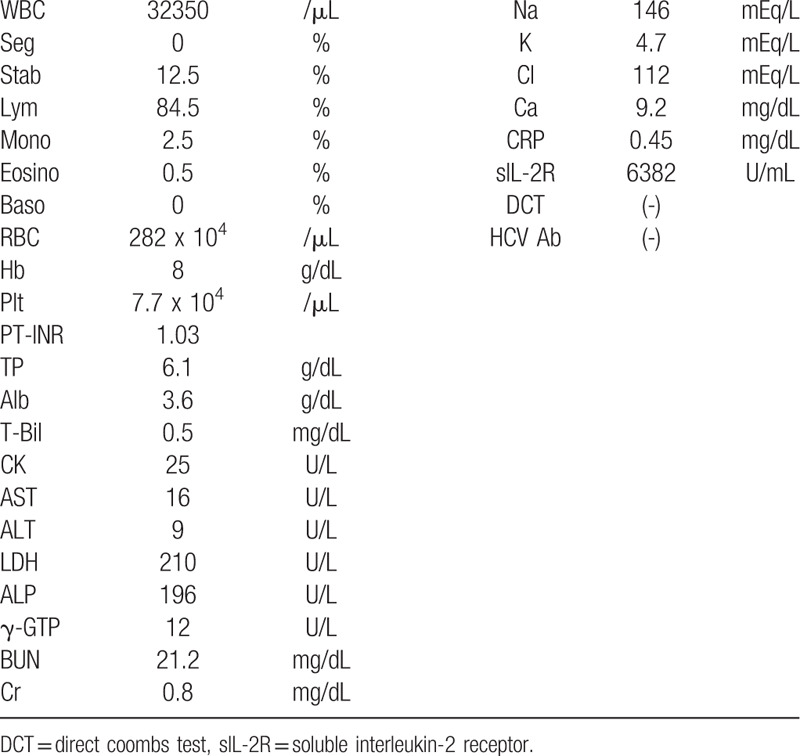
Laboratory data on admission.

**Figure 4:**
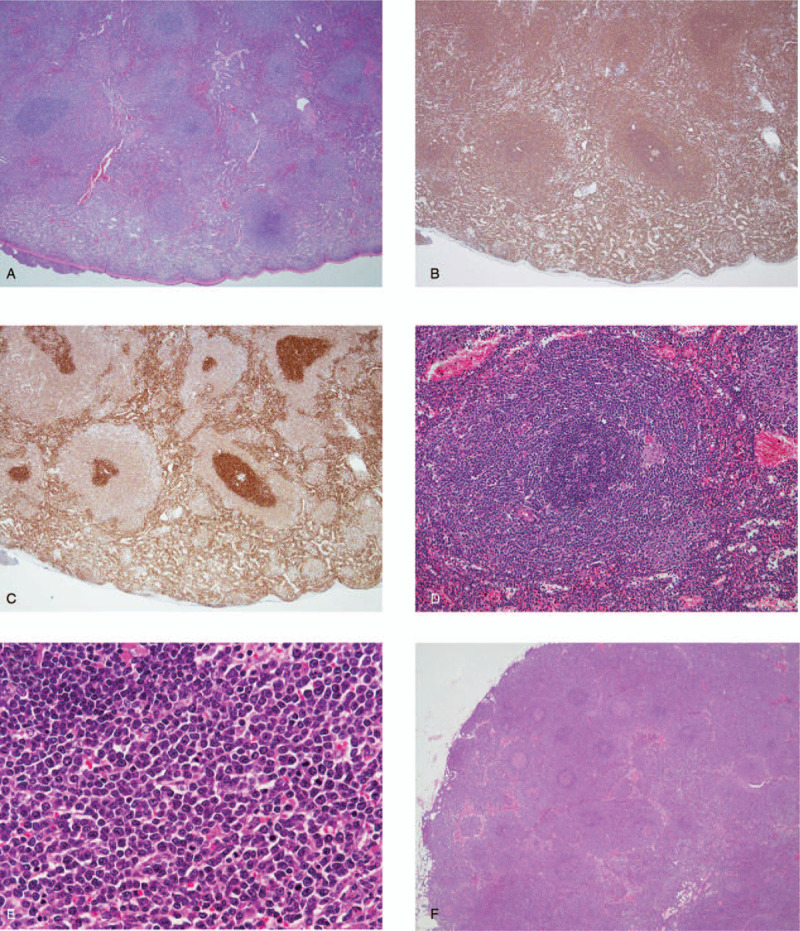
Pathological analysis of the spleen. (A-C) Infiltration of lymphoma cells into the spleen. [(A) Hematoxylin and eosin (H&E) staining (20×), (B) CD20 staining (20×), (C) BCL-2 staining (20×)]. (D) and (E) Infiltration of small and medium lymphoma cells into the marginal zone. [(D) H&E staining (100×), (E) H&E staining (400×)]. (F) Infiltration of lymphoma cells into a splenic hilar lymph node. [H&E staining (20×)].

**Figure 5:**
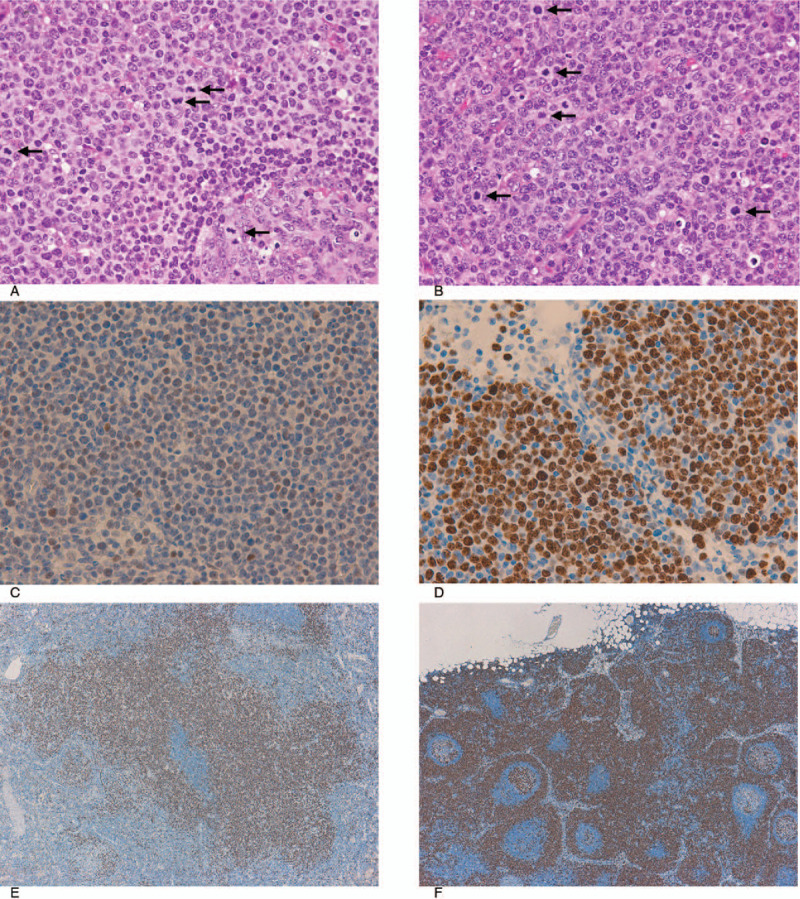
Pathological findings of aggressive variant of splenic marginal zone lymphoma. (A-D) Increased numbers of large lymphoma cells within a splenic hilar lymph node. The arrows of (A) and (B) indicate mitotic cells [(A) and (B) H&E staining (400×), (C) p53 staining (400×), (D) Ki-67 staining (400×)]. (E) and (F) Ki-67 staining in the spleen (E) and a splenic hilar lymph node (F) (40×).

To make an accurate diagnosis, a splenectomy was performed. It revealed micronodular proliferation with enlargement of the marginal zone in the white pulp and infiltration of the red pulp cords and sinusoids by lymphoma cells. Medium-sized lymphoma cells infiltrated the white and red pulp, while small cells invaded mainly the inner core of the white pulp (Fig. 4A-E). Both small and medium cells were positive for CD20, CD22, CD79a, FMC7, and BCL-2, and were negative for CD3, CD5, CD10, CD23, CD25, CD103, and BCL-6 (Fig. 4B and C). G-band analysis showed a complex karyotype (46, X, add (X) (p22.1), add (3) (q27), -6, +12, add (12) (p11.2), add (14) (p11.2)). Fluorescent in situ hybridization (FISH) analyses for IgH/CCND1 and IgH/BCL2 were negative. The splenic hilar lymph nodes were similarly involved (Fig. 4F). Based on these findings, SMZL was considered. On the other hand, we also detected a moderate increase in large-sized abnormal lymphocytes in both the spleen and splenic hilar lymph nodes (Fig. 5A and B). Mitotic lymphoma cells were also increased in those areas (Fig. 5A and B). Because we suspected the tumor was aggressive, we stained the specimens for p53 and Ki-67. Although staining for p53 was negative or weakly positive, staining for Ki-67 was positive in both the large cells and the medium cells (Fig. 5C-F). We therefore made a diagnosis of AV-SMZL. The clinical stage was determined to be IVA (Ann Arbor classification), and the international prognostic index (IPI) was 2 points (low intermediate risk).

To further clarify the molecular basis of the current case, we performed a targeted gene mutational analysis using next-generation sequencing, which revealed mutations in *NOTCH2, NCOA4, PTEN*, *EPHA3,* and *KMT2D* (Table 2). With the exception of those in *KMT2D*, the single nucleotide variation (SNV) mutations in these genes were deemed to be pathogenic based on the FATHMM-XF analysis.

**Table 2:**
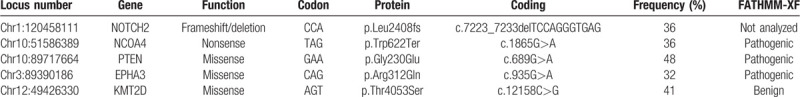
Mutations detected in lymphoma cells.

**Figure 6:**
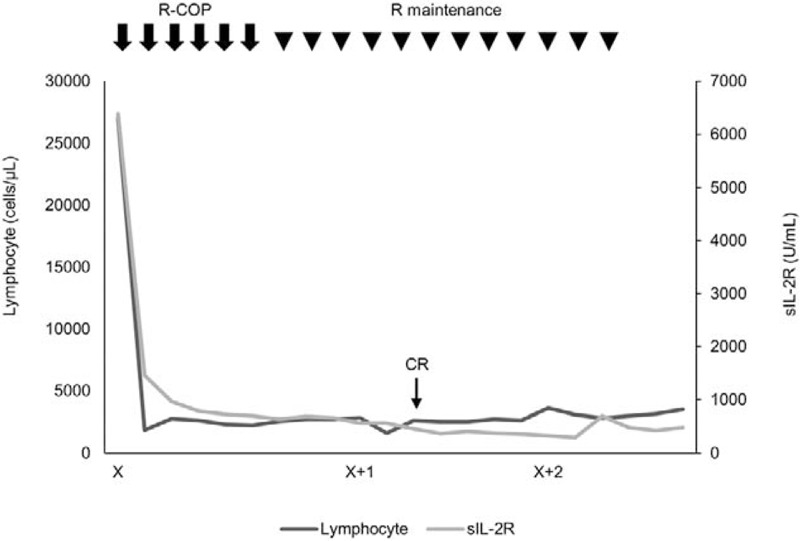
The clinical course of the patient.

We treated the patient with R-COP (rituximab, cyclophosphamide, vincristine, and prednisolone). After 2 cycles of chemotherapy, the abnormal lymphocytes had disappeared from the peripheral and bone marrow blood. However, moderate elevation of serum soluble interleukin-2 receptor levels persisted after 6 cycles of chemotherapy (Fig. 6). We then added 12 cycles of R–maintenance therapy (Fig. 6), and a CR was achieved after 5 cycles of that therapy. Thus far, the patient continues to exhibit a CR.

## 4 Discussion

SMZL is a B-cell neoplasm composed of small lymphocytes that surround and replace the splenic white pulp germinal centers, efface the follicle mantle, and merge with the marginal zone of larger cells. In addition, both small and larger cells infiltrate the red pulp.^[9]^ Lymphoma cells with villous projections are frequently found in the peripheral blood, though there are cases, like this one, without villous lymphocytes.^[10]^ The rates of CD19, CD20, and BCL-2 positivity are high in SMZL.^[11]^ On the other hand, AV-SMZL is a rare and provisional entity.^[1–5]^ Although scattered transformed blasts are often detected in SMZL,^[9]^ AV-SMZL is related to an increase in large blastoid cells, high Ki-67 positivity rates and *TP53* abnormalities.^[1–5]^ In the present case, both an increase of large abnormal lymphocytes and a high Ki-67 positivity rate indicated a diagnosis of AV-SMZL. The very high serum soluble interleukin-2 receptor level and the moderate increase in mitotic cells were consistent with the aggressiveness of this case, while the left leg pain may have been associated with acute enlargement of spleen, as it disappeared shortly after the splenectomy.

To unravel the molecular characteristics of AV-SMZL, we performed targeted gene sequencing analysis. Thanks to recent advances in genome sequencing, cancer panel tests have emerged as useful tools for making diagnoses, predicting prognoses, and determining treatment strategies in rare cancers.^[6]^ In the present case, we found mutations in *NOTCH2, NCOA4, PTEN*, *EPHA3,* and *KMT2D*, whereas *TP53* mutation was not detected, which is consistent with the negative or only weakly positive staining for p53.^[3]^ FATHMM-XF analysis predicted the single nucleotide mutations detected in *NCOA4, PTEN,* and *EPHA3* to be pathogenic (Table 2). *NOTCH2* is one of the most frequently (10% to 25%) mutated genes in SMZL,^[12]^ whereas *NOTCH2* mutations are not found in chronic lymphocytic leukemia (CLL), mantle cell lymphoma (MCL), low grade follicular lymphoma (FL), or hairy cell leukemia (HCL).^[13,14]^ The frameshift/deletion mutation we found in this case affects the C-terminal PEST domain of NOTCH2, which is critical for NOTCH2 proteasomal degradation. This mutation is predicted to delete that domain.^[15]^ While *NCOA4, PTEN,* and *EPHA3* mutations have not been reported in SMZL, the COSMIC (catalogue of somatic mutations in cancer) database (https://cancer.sanger.ac.uk/cosmic) shows that c.689G > A mutation of *PTEN* occurs in glioma, and c.935G > A mutation of *EPHA3* occurs in colon, liver, and prostate cancers. These findings suggest that the genetic profile of this case supports the diagnosis of SMZL and that mutation of *NCOA4, PTEN,* and *EPHA3* may be associated with the aggressiveness and persistence of AV–SMZL.

Despite its poor prognosis, there is no standard treatment for AV-SMZL. We treated the patient with 6 cycles of R-COP therapy and 12 cycles of R-maintenance therapy. In earlier studies of SMZL, rituximab mono or combination therapies (CR rates of 42%–79%) were more effective than other treatments without rituximab (CR rates of 18%–30%).^[16,17]^ Moreover, rituximab-maintenance therapy extended freedom from progression (FFP) in SMZL and was effective even in the patients exhibiting only a partial response (PR) after induction therapy.^[18]^ This suggests the treatment administered to this case was reasonable, though careful monitoring for disease relapse is necessary.

In conclusion, this is a case of AV-SMZL in which a cancer panel test successfully detected genetic alterations that are potentially associated with its pathogenesis. These findings suggest that genetic analysis is useful for making diagnoses as well as for determining treatment strategies in AV-SMZL.

## Acknowledgments

The authors thank Dr William F. Goldman for editing the manuscript.

## Author contributions

K.I., T.T. and T.I. designed the study. K.I., Y.T. and Y.M. performed the experiments. K.I., Y.S., T.N. and T.T. performed the data analysis. K.I., H.S. and T.H. wrote the manuscript. All authors reviewed and approved the final manuscript.
